# Draft Genome Sequence of the Green Alga Scenedesmus acuminatus SAG 38.81

**DOI:** 10.1128/MRA.01278-19

**Published:** 2020-06-11

**Authors:** Yekaterina Astafyeva, Malik Alawi, Daniela Indenbirken, Dominik Danso, Adam Grundhoff, Dieter Hanelt, Wolfgang R. Streit, Ines Krohn

**Affiliations:** a Universität Hamburg, Institute of Plant Science and Microbiology, Department of Microbiology and Biotechnology, Hamburg, Germany; b Department of Aquatic Ecophysiology and Phycology, Biocenter Klein Flottbek, University of Hamburg, Hamburg, Germany; c University Medical Center Hamburg-Eppendorf, Bioinformatics Core, Hamburg, Germany; d Heinrich-Pette-Institute, Leibniz Institute for Experimental Virology, Virus Genomics Research Unit, Hamburg, Germany; University of California, Riverside

## Abstract

Scenedesmus acuminatus, also known as Tetradesmus acuminatus, is a promising green microalga for sustainable production of microalga products, including valuable compounds such as astaxanthin, β-carotene, and lutein, polysaccharides such as β-glucan, and polyunsaturated fatty acids. Here, we report the draft whole-genome sequence of *Scenedesmus acuminatus* SAG 38.81.

## ANNOUNCEMENT

Microalgae are considered potential cell factories for manufacturing high-value products such as food supplements and bioactive compounds, as well as low-value products, including biofuels (1, 2). These characteristics are present in freshwater microalga *Scenedesmus* spp., whose cultivation has a satisfactory cost-benefit ratio and generates high-quality biomass (3). Scenedesmus acuminatus is a green alga in the family Scenedesmaceae, a member of the Chlorophyceae (4). The observed high carbohydrate and lipid accumulation in this *Scenedesmus* microalga signifies its potential for bioethanol and biodiesel production (5, 6). *Scenedesmus* species can be a rich source of polysaccharides, polyunsaturated fatty acids, and pigments, including lutein (7), a pigment that is recommended for the prevention of some types of cancer and cardiovascular disease (8, 9).

*Scenedesmus acuminatus* SAG 38.81 was cultivated in BG11 medium at 20 ± 1°C and 100 ± 10 μmol photons m^−2^ s^−1^ with a 14/10-h light/dark period. Total genomic DNA was extracted for a detailed genomic analysis using the NucleoBond high-molecular-weight genomic DNA kit for microorganisms (Macherey-Nagel, Germany) following the manufacturer’s instructions and a previously published enzymatic cell lysis protocol with some modifications, including freezing in liquid nitrogen, bead beating, and an additional lysis pretreatment with proteinase K and lysozyme for 24 h at 55°C (10). The extracted DNA was sequenced on an Illumina NextSeq 500 platform using rapid sequencing by synthesis (SBS) chemistry v2 (Illumina, San Diego). For this, the library was constructed applying the NEBNext Ultra DNA library prep kit for Illumina (New England Biolabs) according to the manufacturer’s protocol. The sample was sequenced on the NextSeq 500 platform (2 × 150 bases) with approximately 290 million reads.

The same extracted DNA that was sequenced on the Illumina platform was additionally sequenced using a MinION device (Oxford Nanopore Technologies [ONT], Oxford, UK). The library was prepared using the PCR-free rapid barcoding kit (SQK-RBK004) according to the manufacturer’s protocol. An optional cleanup step using magnetic beads (AMPureXP, Beckman Coulter) was performed, and the barcoded DNA was subsequently loaded on an R9.4 flow cell and sequenced with 326,000 reads. The genome is covered on average 19.66 times.

Sequencing adapters and low-quality (Phred score below 20) bases were removed from the 3′ end of the sequencing reads using Trimmomatic v0.38 (11). Sequences shorter than 35 bp after trimming were discarded.

Short reads (Illumina) and long reads (ONT) were used for a hybrid genome assembly with MaSuRCA v3.3.3 (12). The ﬁnal draft assembly consists of 119,154,196 bp with a GC content of 54%. The final genome assembly resulted in 2,954 contigs (*N*_50_, 87,177 bp; *L*_50_, 370), with a largest contig size of 730,299 bp.

The presented draft nuclear genome assembly of *Scenedesmus acuminatus* SAG 38.81 provides a strong basis for comparative genetic analyses and will help elucidate metabolic processes.

### Data availability.

The raw sequence and assembly data generated during this study have been submitted to the European Nucleotide Archive (ENA). They are publicly available under accession no. PRJEB34756 and BioSample no. SAMEA6056938.
